# Pharmacological and Phytochemical Insights Into *Ficus benghalensis* (Indian Banyan): Anti‐Inflammatory, Antioxidant, and Anticancer Potentials

**DOI:** 10.1002/fsn3.71982

**Published:** 2026-06-05

**Authors:** Zainab Ali, Farhang Hameed Awlqadr, Uswa Ali, Iffat Ullah, Muhammad Tayyab Arshad, Abdul Rauf, Humaira Parveen, Sayeed Mukhtar, Uzma Faridi, Md. Sakhawot Hossain

**Affiliations:** ^1^ Department of Biochemistry University of Agriculture Faisalabad Faisalabad Pakistan; ^2^ Food Science and Quality Control, Halabja Technical College Sulaimani Polytechnic University Sulaymaniyah Iraq; ^3^ Department of Pharmaceutical Chemistry, Faculty of Pharmaceutical Sciences Prince of Songkla University Hat Yai Songkhla Thailand; ^4^ Drug Delivery System Excellence Center, Faculty of Pharmaceutical Sciences Prince of Songkla University Hat Yai Songkhla Thailand; ^5^ Functional Food and Nutrition Program, Faculty of Agro‐Industry Prince of Songkla University Hat Yai Songkhla Thailand; ^6^ Organic and Medicinal Chemistry Research Lab, Department of Chemistry, Faculty of Science University of Tabuk Tabuk Saudi Arabia; ^7^ Department of Biochemistry, Faculty of Science University of Tabuk Tabuk Saudi Arabia; ^8^ Department of Nutrition and Food Technology Jashore University of Science and Technology Jashore Bangladesh; ^9^ Department of Nutrition and Food Engineering Daffodil International University Dhaka Bangladesh

**Keywords:** anticancer, antidiabetic, anti‐inflammatory, antimicrobial, antioxidant, *F. benghalensis*, phytochemicals

## Abstract

*Ficus benghalensis* L. (Indian banyan), a prominent member of the Moraceae family, is a widely utilized ethnomedicinal plant with substantial therapeutic relevance. This comprehensive review critically synthesizes recent phytochemical, pharmacological, and mechanistic evidence supporting the antioxidant, antimicrobial, antidiabetic, anti‐inflammatory, and anticancer potentials of *F. benghalensis* . Phytochemical investigations reveal a rich diversity of bioactive constituents, including flavonoids (quercetin, kaempferol, apigenin, and leucopelargonidin), terpenoids (lupeol, α‐ and β‐amyrin acetates, and ursolic acid), sterols (β‐sitosterol and stigmasterol), phenolic acids, alkaloids, coumarins, glycosides, and fatty acid derivatives distributed across different plant parts. Strong antioxidant activity is reflected in the low IC_50_ values in DPPH, ABTS, FRAP, and CUPRAC assays, correlating with high phenolic and flavonoid content and possible modulation of redox‐regulatory pathways. Antimicrobial studies report broad‐spectrum antibacterial and antifungal activity with notable zones of inhibition and minimum inhibitory concentrations, particularly in bark and leaf extracts. Antidiabetic effects are mediated through inhibition of α‐amylase, α‐glucosidase, aldose reductase, and protein tyrosine phosphatase 1B (PTP1B), enhancement of glucose uptake via GLUT‐2, and activation of the PI3K/Akt signaling pathway, supported by in silico, in vitro, and in vivo evidence. Anti‐inflammatory activity is substantiated by significant inhibition of COX‐1, COX‐2, 5‐LOX, and pro‐inflammatory cytokines (IL‐6 and IL‐1β), alongside suppression of EGFR/PI3K/Akt signaling by a novel fatty acid glucoside. Anticancer potential is evidenced by selective cytotoxicity, induction of apoptosis via caspase activation and topoisomerase I inhibition, oxidative stress generation, and G_2_/M cell cycle arrest across multiple cancer cell lines. Nanoformulation approaches further enhance bioactivity and selectivity, although mechanistic clarity remains limited. Despite robust preclinical evidence, gaps persist in pharmacokinetics, standardization, toxicity profiling, and clinical validation. Overall, this review bridges traditional knowledge with modern molecular pharmacology, highlighting *F. benghalensis* as a promising multi‐target phytotherapeutic candidate for future drug discovery and development.

## Graphical abstract

The aim of this review is to critically evaluate the therapeutic potential of *Ficus benghalensis* L. by integrating ethnomedicinal knowledge with contemporary scientific evidence. The objectives are to summarize its phytochemical profile, analyze antioxidant, antimicrobial, antidiabetic, anti‐inflammatory, and anticancer activities, elucidate underlying molecular mechanisms, and identify research gaps regarding standardization, pharmacokinetics, toxicity, and clinical translation.

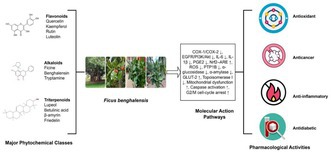

## 1 Introduction

The genus *Ficus*, belonging to the family Moraceae, represents one of the largest and most diverse genera of flowering plants, comprising over 800 recognized species distributed across tropical and subtropical regions (Yang et al. 2023). These species are widely found in Asian countries such as Malaysia, India, and Nepal, and are well‐known for their rich phytochemical composition and diverse pharmacological potential (Amri et al. 2024). Numerous studies have revealed that Ficus species contain a broad spectrum of bioactive compounds, including phenols, flavonoids, alkaloids, tannins, saponins, terpenoids, glycosides, steroids, and essential oils, which contribute to their therapeutic versatility and significance in traditional medicine systems (Murugesu et al. 2021; Li, Lu, et al. 2024). Among these, *F. benghalensis* L., commonly known as the Indian banyan, holds exceptional medicinal, ecological, and cultural importance. This large evergreen tree, reaching heights of 20–30 m, develops aerial roots that form massive secondary trunks, allowing it to dominate open forest spaces (Chaudhary et al. 2015; Sanap and Shisode 2025). Beyond its ecological role, the species is deeply revered in Indian culture and Hindu mythology, symbolizing longevity and immortality (Babaeva et al. 2025; Basnett et al. 2023). As the national tree of India, *F. benghalensis* is commonly planted near temples, gardens, and roadsides for both its spiritual significance and its expansive shade.

Phytochemical investigations of *F. benghalensis* have demonstrated the presence of numerous secondary metabolites such as phenols, flavonoids, alkaloids, tannins, saponins, terpenoids, glycosides, and steroids. However, only a few specific compounds, such as β‐sitosterol, stigmasterol, and lupeol, have been isolated and characterized from the bark and aerial roots (Tahir et al. 2023; Das et al. 2022; Liu et al. 2025; Sánchez‐Hoyos et al. 2024). The latex of the tree is rich in caoutchouc, resin, and albuminous substances, which contribute to its healing and antimicrobial properties. In traditional Indian medicine systems such as Ayurveda and Unani, nearly all parts of the tree, including the roots, bark, leaves, fruits, and latex, are therapeutically utilized. Decoctions of the bark and roots are traditionally prescribed for ailments such as diabetes, diarrhea, dysentery, and rheumatism, while the latex is used for wound healing and blood purification. Leaves are applied externally for ulcers and skin disorders, and fruits are consumed to aid digestion and support female reproductive health, emphasizing the plant's broad ethnomedicinal applications (Khadivi et al. 2022; Kumar et al. 2014; Chaudhary et al. 2023).

Modern pharmacological studies have validated several traditional claims, revealing a wide range of bioactivities associated with *F. benghalensis* extracts. The plant exhibits potent antioxidant properties due to its high phenolic and flavonoid content, which mitigates oxidative stress linked to cardiovascular, metabolic, and neurodegenerative disorders (Basnett et al. 2023; Morante‐Carriel et al. 2024). Preclinical and clinical studies have also demonstrated significant antidiabetic activity, with bark and root extracts improving glucose tolerance, insulin secretion, and lipid metabolism (Ahmad et al. 2023; Garas et al. 2020). Furthermore, the plant's anti‐inflammatory potential through inhibition of pro‐inflammatory mediators supports its traditional use in treating arthritis and rheumatism. *F. benghalensis* has also shown antimicrobial efficacy against bacterial and fungal pathogens, along with emerging evidence of anticancer and antitumor effects attributed to terpenoids and flavonoids that induce apoptosis and inhibit tumor proliferation. Additional studies highlight its hepatoprotective, immunomodulatory, wound‐healing, anticoagulant, and antistress properties, reflecting its multifaceted pharmacological significance (Murugesu et al. 2021; Li, Lu, et al. 2024; Sánchez‐Hoyos et al. 2024).

**FIGURE 1:**
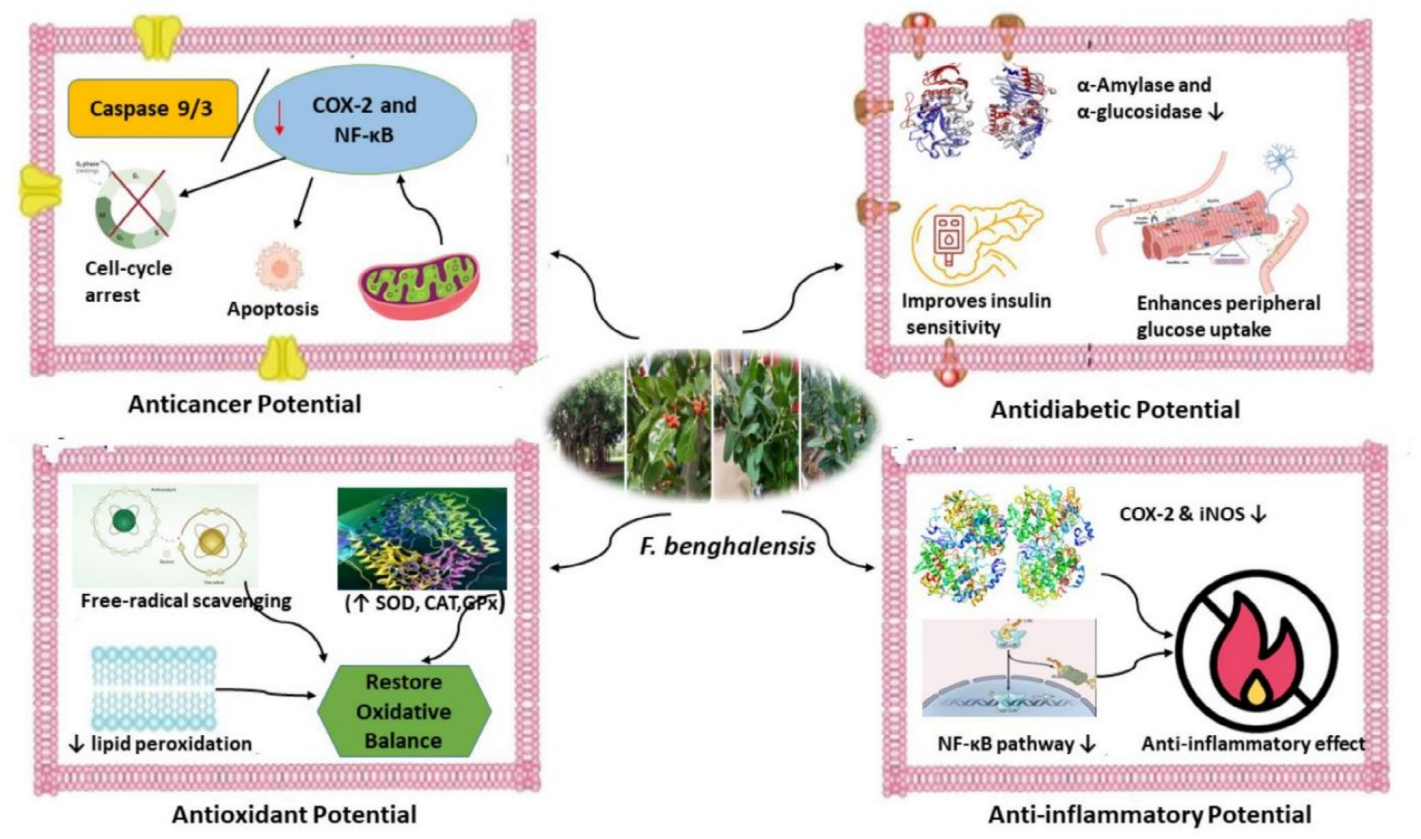
Molecular mechanisms underlying the biological activity of the various constituents of *F. benghalensis* . It shows molecular mechanisms underlying the biological activity of the various constituents of *F. benghalensis* . (1) The constituents possess anti‐inflammatory activity via selective COX‐1/COX‐2 inhibition and suppression of the EGFR/PI3K/Akt pathway, which leads to the inhibition of IL‐6, IL‐1β, and PGE_2_. (2) Constituent of the plant shows potential antioxidant activity by the modulation of the Nrf2‐ARE pathway and scavenging of free reactive oxygen species (ROS). (3) The plant shows antidiabetic activity via the inhibition of PTP1B, the enhancement of glucose transport via GLUT‐2, and the inhibition of α‐glucosidase and α‐amylase. (4) The plant shows anticancer properties through the inhibition of topoisomerase I, mitochondrial dysfunction, the triggering of apoptotic pathways via caspase activation, and the arrest of G2/M cell cycle. The green arrows are for activation, and the red arrows are for inhibition (1).

Despite its therapeutic potential, certain safety considerations and research limitations persist. Concentrated latex and bark extracts may cause gastrointestinal discomfort or hypersensitivity in sensitive individuals. Although *F. benghalensis* is generally safe at therapeutic doses, standardized extraction methods, dose optimization, and comprehensive clinical trials are still lacking. Furthermore, mechanistic insights into its bioactive compounds and molecular targets remain incomplete. Addressing these research gaps through well‐designed experimental and clinical studies could enhance understanding of its pharmacological mechanisms and facilitate the development of standardized herbal formulations (Chakraborty et al. 2022; Ghimire et al. 2020). In this review, we provide an updated synthesis of the pharmacological activities and phytochemical constituents of *F. benghalensis* , emphasizing its antioxidant, antidiabetic, anti‐inflammatory, anticancer, and wound‐healing properties. This overview aims to bridge traditional ethnomedicinal knowledge with modern pharmacological evidence, contributing to future research efforts focused on the discovery of novel therapeutic agents for the prevention and management of chronic diseases. Figure 1 shows the mechanistic overview of the pharmacological potential of *F. benghalensis* .

Current phytomedicine research faces major limitations due to the use of non‐standardized crude extracts, inconsistent bioactivity reporting, lack of pharmacokinetic validation, minimal clinical translation, and insufficient safety and herb–drug interaction studies. Recent reviews by Singh, Dhankhar, Kapoor, and Sharma (2023) and Tahir et al. (2023) have described the ethnomedicinal and general pharmacological uses of *F. benghalensis* . Addressing these gaps, the present review on *F. benghalensis* provides a comprehensive and updated synthesis by systematically compiling quantitative pharmacological data, including IC_50_, MIC, and dose–response values, while integrating molecular‐level mechanisms such as COX‐1/COX‐2 inhibition and EGFR/PI3K/Akt‐mediated apoptosis. In addition, the review expands the therapeutic scope by evaluating green nanotechnology applications that enhance bioactivity, offering a critical comparison of anticancer efficacy and selectivity across cell lines, and incorporating recent advances in computational pharmacology to elucidate multi‐target actions related to antidiabetic and anticancer effects, thereby strengthening the translational relevance of *F. benghalensis* in evidence‐based drug discovery.

### 1.1 Methodology

To gather a critical review of the phytochemistry, pharmacological trials, traditional uses, and the toxicity of *F. benghalensis* , a systematic literature review was performed. Major scientific databases were searched, such as Google Scholar, ScienceDirect, PubMed Central, Web of Science, Scopus, and ResearchGate, with specific keyword sets like *F. benghalensis* , phytochemistry, pharmacological activities, anticancer, antioxidant, antimicrobial, antidiabetic, ethnomedicine, bioactive compounds, and toxicology.

Methodological rigor and relevance were attained by strict inclusion and exclusion criteria. Inclusion criteria were English‐language publications before 2024; original experimental studies (in vitro, in vivo, and ex vivo and limited clinical studies), comprehensive review articles, and comparative studies relative to related Ficus species having well‐reported methodologies and quantitative data. Articles that are non‐methodologically detailed, duplicate, unverified assertions, non‐peer‐reviewed sources, abstracts of conferences, and dissertation works were eliminated.

The screening was conducted in two phases. First, there was an assessment of the title and abstract, and finally, a full‐text evaluation was done to determine the quality of science, reliability of data, and relevance to pharmacological mechanisms. Pharmacological divisions were categorized to give preclinical, animal, and clinical findings. There was a preference for studies with strong quantitative parameters (IC_50_, EC_50_, and MIC), dose–response associations, statistical tests, quantitative standard assays, and proper controls.

Brief screening of patent databases, which were not used as primary evidence, helped put bioactive applications into context. More than 100 sources, mainly original research, have been incorporated to ensure the coverage is not limited to secondary sources to a significant extent. This systematic method allowed forming a critical synthesis of evidence that was available, unearthing mechanistic knowledge, and also pointing out research gaps.

## 2 Botanical Description of *F. benghalensis*

**FIGURE 2:**
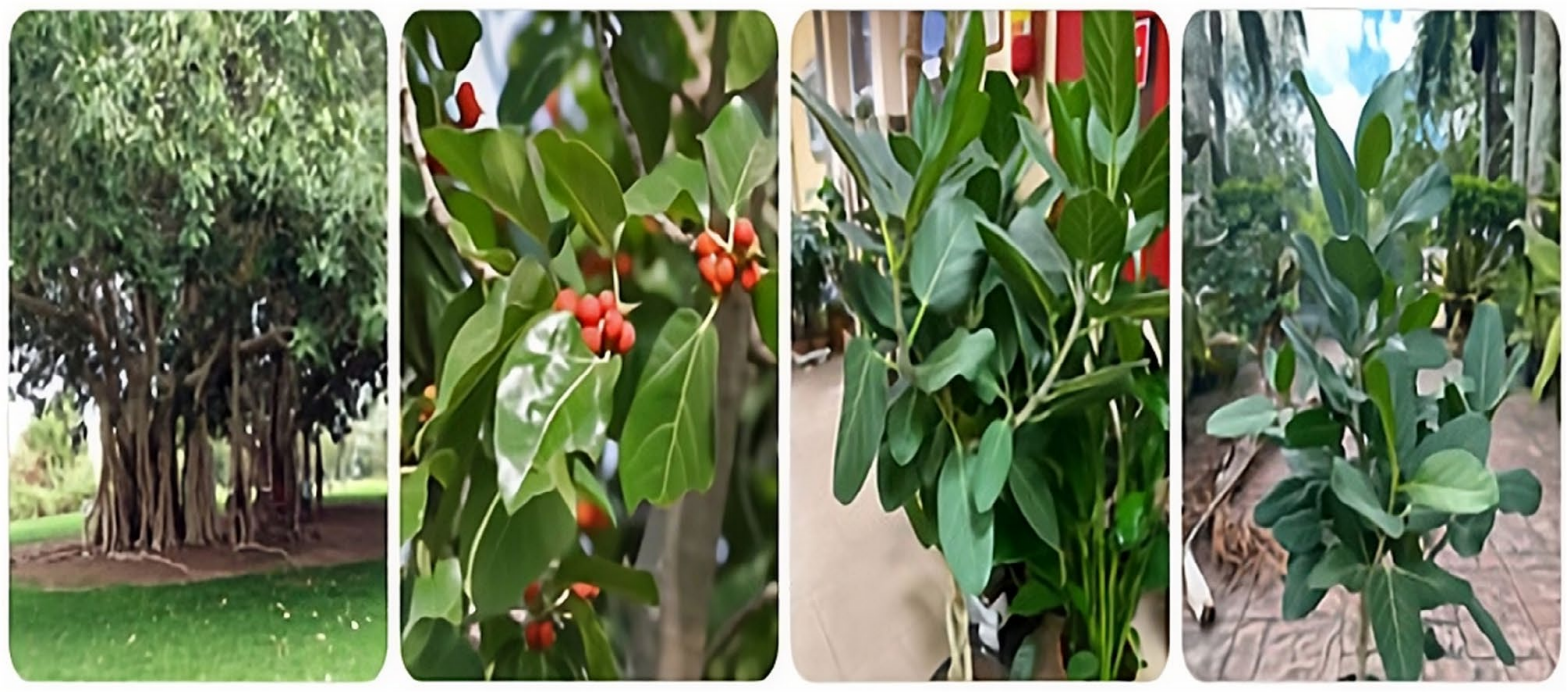
Botanical description of *F. benghalensis* showing key morphological features: whole tree, leaf, and syconium (fruit).

*F. benghalensis* L., a perennial species of the family *Moraceae*, is widely distributed across South Asia, particularly in India, Sri Lanka, and Pakistan. Deeply integrated into the region's cultural and religious traditions, it is commonly cultivated in sacred and open spaces (Verma 2016). This keystone species exhibits remarkable morphological adaptations, including epiphytic germination and the development of aerial prop roots that undergo secondary thickening to form pillar‐like trunks, enabling extensive horizontal spread and large canopy formation (Deepa et al. 2018). The tree's coarse, glossy, ovate‐elliptic leaves form a dense crown, while its unique reproductive structure, the syconium, encloses numerous male and female flowers within a specialized inflorescence (Murugesu et al. 2021). Figure 2 shows the Botanical description of *F. benghalensis* showing key morphological features: Whole tree, Aerial roots, Leaf, and Syconium (fruit). Overall, *F. benghalensis* demonstrates exceptional morphological, reproductive, and ecological adaptations that contribute to its ecological dominance and cultural importance. (Singh 1979). Table 1 describes botanical and ecological features of *F. benghalensis* .

**TABLE 1:** Botanical and ecological features of *F. benghalensis* .

Feature	Description	References
Family	Moraceae (mulberry family)	Verma (2016)
Growth Habit	Evergreen, epiphytic origin, extensive aerial roots, and multiple trunks	Moustafa (2020)
Height	20–30 m or more	Moustafa (2020)
Leaf	Ovate/elliptic, leathery, 10–20 cm long, clustered at branch ends	Moustafa (2020)
Inflorescence	Syconium with male and female flowers, pollinated by fig wasps	Moustafa (2020)
Fruit	Small, red/purple figs, 1–2 cm diameter, in leaf axils	Moustafa (2020)
Habitat	Tropical/subtropical, humid, moist soils, and drought‐resistant	Singh (1979)
Notable feature	Aerial roots form new trunks, and a vast canopy spreads	Moustafa (2020)

## 3 Phytochemical Constituents of *F. benghalensis*

### 3.1 Flavonoids

*F. benghalensis* contains a rich array of flavonoids, mainly in leaves, bark, and roots. Key compounds include quercetin (in leaves and bark) and rutin, well‐known antioxidants and anti‐inflammatories (Murugesu et al. 2021). Kaempferol (in methanolic extracts) and apigenin (especially abundant) have antidiabetic and anti‐inflammatory effects. Importantly, bark and aerial roots yield leucoanthocyanidins (e.g., *leucopelargonidin* glycosides) that are potent antioxidants (Sahu et al. 2024). An unusual flavone, *3,4′,5,7‐tetrahydroxy‐3′‐methoxyflavone*, has been identified and shown to selectively inhibit PTP1B (a diabetes target) (Khanal and Patil 2019). Overall, these flavonoids scavenge free radicals and inhibit carbohydrate‐hydrolyzing enzymes, helping explain the plant's antioxidant and antidiabetic activity (Murugesu et al. 2021).

### 3.2 Sterols

The main phytosterols in *F. benghalensis* are β‐sitosterol and stigmasterol, particularly in bark and aerial roots (Verma et al. 2015). β‐Sitosterol (isolated from roots/bark) has documented anti‐inflammatory effects. Stigmasterol (present at high levels) also reduces inflammation dose‐dependently (Bhaskara Rao et al. 2014). Notably, stigmasterol acetate (an acetylated derivative) is even more active in vivo: in rodent edema models, 100 mg/kg stigmasterol acetate gave 42.5% inhibition of inflammation (Mazumder et al. 2022). These sterols likely contribute to the reported anti‐inflammatory and lipid‐lowering actions of the plant.

### 3.3 Terpenoids

*F. benghalensis* is rich in triterpenes and terpenes. Lupeol (a triterpene) is especially abundant (17.4% of leaf extract); it has anti‐inflammatory and hepatoprotective effects (Singh, Dhankhar, Kapoor, and Sharma 2023). Its acetate derivative also shows strong bioactivity. Other major triterpenoids include friedelin, friedelanol, betulinic acid, and amyrins. For example, β‐amyrin and α‐amyrin (and their acetates) were isolated from aerial roots; these amyrins are known analgesics and anti‐inflammatories. In vivo, α‐amyrin acetate (100 mg/kg) inhibited rat paw edema by 62.5%, and β‐amyrin acetate by 45%. Minor terpenes include lup‐20 (29)‐en‐3‐one (20.5% of fruit extract) and sesquiterpenes like phytol (identified by GC–MS) (Singh, Dhankhar, Kapoor, and Sharma 2023; Patil and Patil 2010). These terpenoids likely underlie many of the bark and leaf extracts' pharmacological effects.

### 3.4 Phenolic Acids

The plant contains several simple phenolics. Gallic acid is reported and contributes strong antioxidant activity. GC–MS of fruit shows gallic, caffeic, chlorogenic, coumaric, ferulic, and ellagic acids, all known antioxidants and enzyme inhibitors (Jayasree Radhakrishnan and Venkatachalam 2020; Tharini et al. 2018). Notably, 3,4‐dihydroxybenzoic acid (salicylic acid derivative) was identified by LC–MS and acts as an α‐amylase inhibitor. Among fatty acids, α‐linolenic acid (18:3), listed as octadecatrienoic acid, comprises 15.2% of fruit oil, while linoleic (18:2) and palmitic acids are also present (Singh, Dhankhar, Kapoor, and Sharma 2023; Faysal et al. 2023; Tkachenko et al. 2017). A high drug‐likeness is predicted for 4‐methoxybenzoic acid, another minor phenolic. These acids add to the plant's antioxidant and metabolic regulatory effects.

### 3.5 Alkaloids

Various alkaloids have been detected in leaves, bark, fruit, and aerial roots. Although their exact structures are not fully elucidated, these alkaloids exhibit neuroprotective and antimicrobial activities (Murugesu et al. 2021; Khaliq 2017). In preliminary screens, bark and root extracts showed alkaloid‐like reactions, and such fractions suppressed microbial growth (Mazumder et al. 2018). Thus, alkaloids likely contribute to *F. benghalensis* 's broad pharmacological profile, particularly in the nervous system and infection models.

### 3.6 Coumarins

*F. benghalensis* contains furanocoumarins. Bergapten (5‐methoxypsoralen) and psoralen are reported especially in the seed oil and to some extent in leaves (Bhaskara Rao et al. 2014; Faiyaz Ahmed et al. 2011). These compounds are photoreactive: psoralen is a known photosensitizer, and bergapten has anti‐inflammatory/neuroprotective effects (e.g., inhibits AChE, improves cognition in models) (Senol et al. 2011; Orhan et al. 2008; Dincel et al. 2013). Their presence suggests caution (potential phototoxicity) but also may underlie some traditional uses.

### 3.7 Novel Bioactive Glycosides

Recent studies have identified unique glycosides in *F. benghalensis* . Bengalenoside is a phenolic glycoside from leaves that shows hypoglycemic (antidiabetic) activity (Tahir et al. 2023). Carpachromene, a stilbene‐like compound from leaves, has potent antiproliferative effects: it induces G2/M cell‐cycle arrest and apoptosis in leukemia cells via topoisomerase I inhibition (Hassan et al. 2022). Another newly reported compound is a fatty‐acid glucoside from leaves: it strongly inhibits LPS‐induced inflammation in macrophages by blocking COX enzymes and binding EGFR to suppress PI3K/Akt signaling (Alaaeldin et al. 2022). These novel molecules illustrate the plant's unique chemistry and may serve as leads for drug development.

### 3.8 Other Compounds

In addition to the above classes, *F. benghalensis* contains a variety of other phytochemicals (Table 2). For example, tocopherols (vitamin E) were detected (3.9% of leaf extract) (Singh, Dhankhar, Kapoor, and Sharma 2023), contributing antioxidant protection. The monoterpene carvacrol has been reported in GC–MS surveys (imparting antimicrobial and antioxidant activity) (Zangeneh et al. 2019). A simple benzophenone derivative has also been tentatively identified. Furthermore, the plant is rich in saponins, tannins, and anthraquinones, which are common in many Ficus species. For instance, stem bark extracts contain various tannins (leucopelargonidin glycosides) (Cherian and Augusti 1993). Together, these compounds reinforce the reported antimicrobial, antioxidant, and wound‐healing effects of *F. benghalensis* .

**TABLE 2:** Major phytochemical classes in *F. benghalensis* .

Phytochemical class	Major compounds reported	Plant part (s)	Reference(s)
Flavonoids	Quercetin, rutin, kaempferol, apigenin, leucoanthocyanidins (leucopelargonidin glycosides), and 3,4′,5,7‐tetrahydroxy‐3′‐methoxyflavone	Leaves, bark, roots, and aerial roots (kaempferol mainly in methanolic extracts)	Murugesu et al. (2021), Sahu et al. (2024), and Khanal and Patil (2019)
Sterols	β‐Sitosterol, stigmasterol, and stigmasterol acetate	Bark, roots, and aerial roots	Verma et al. (2015), Bhaskara Rao et al. (2014), and Mazumder et al. (2022)
Terpenoids (mainly triterpenoids)	Lupeol, lupeol acetate, friedelin, friedelanol, betulinic acid, α‐amyrin, β‐amyrin, α‐amyrin acetate, β‐amyrin acetate, lup‐20(29)‐en‐3‐one, and phytol	Leaves, aerial roots, fruits, and bark	Singh, Dhankhar, Kapoor, and Sharma (2023) and Patil and Patil (2010)
Phenolic acids and related simple phenolics	Gallic acid, caffeic acid, chlorogenic acid, *p*‐coumaric acid, ferulic acid, ellagic acid, 3,4‐dihydroxybenzoic acid, and 4‐methoxybenzoic acid	Leaves, fruits (majorly); other parts not specified	Jayasree Radhakrishnan and Venkatachalam (2020) and Tharini et al. (2018)
Fatty acids	α‐Linolenic acid (octadecatrienoic acid, 18:3), linoleic acid (18:2), and palmitic acid (16:0)	Fruits (oil)	Singh, Dhankhar, Kapoor, and Sharma (2023), Faysal et al. (2023), and Tkachenko et al. (2017)
Alkaloids	Uncharacterized alkaloids	Leaves, bark, fruits, and aerial roots	Murugesu et al. (2021), Khaliq (2017), and Mazumder et al. (2018)
Coumarins (furanocoumarins)	Bergapten (5‐methoxypsoralen) and psoralen	Seeds (oil) and leaves	Senol et al. (2011), Orhan et al. (2008), and Dincel et al. (2013)
Glycosides and specialized metabolites	Bengalenoside (phenolic glycoside), carpachromene, and fatty‐acid glucoside	Leaves	Tahir et al. (2023), Hassan et al. (2022), and Alaaeldin et al. (2022)
Other constituents	Tocopherols (vitamin E), carvacrol, benzophenone derivative, saponins, tannins (including leucopelargonidin glycosides), and anthraquinones	Leaves, stem bark (tannins), other parts not specified	Singh, Dhankhar, Kapoor, and Sharma (2023), Zangeneh et al. (2019), and Cherian and Augusti (1993)

Most studies report an enhancement of bioactivity through nanoformulation, but a critical analysis of each case must be conducted. Nayak et al. (2016) synthesized silver nanoparticles (AgNPs) using bark extract and reported dose‐dependent antiproliferative activity on MG‐63 osteosarcoma cells. However, this study did not clarify whether an increase in efficacy, in this case, was due to (1) increased bioavailability of phytochemicals, which then increased cellular uptake, (2) inherent cellular activity of the silver nanoparticles, (3) some cytotoxic effect due to silver nanoparticles, or (4) a combination of all three (Nayak et al. 2016). AgNPs synthesized with *F. benghalensis* extract also encountered the same shortcomings as Singh, Dhankhar, Kapoor, and Sharma (2023), and reported only 90% DPPH inhibition, but did not perform comparative studies with the same concentrations of crude extract. Because of that, it is hard to quantify the degree of enhancement (Singh, Dhankhar, Kapoor, and Sharma 2023). To be clear on the percent enhancement due to nanoformulation in subsequent studies, the authors suggest that, at a minimum, the following be compared: (a) crude extract alone, (b) nanoparticles alone, (c) phytochemical‐loaded nanoparticles, and (d) mixtures of the above physically, while taking care to end up with the same total dose (Singh, Dhankhar, Kapoor, and Sharma 2023).

## 4 Pharmacological Activities of *F. benghalensis*

### 4.1 Antioxidant Activity

*F. benghalensis* is widely recognized for its potent antioxidant properties, attributed to its abundance of phenolics, flavonoids, tannins, and other bioactive compounds. Extensive studies have confirmed that extracts derived from various plant parts, including fruits, leaves, bark, seeds, latex, and aerial roots, exhibit strong free radical scavenging and reducing power, validating their traditional medicinal use for promoting health and preventing oxidative stress–related diseases (Amali et al. 2025). A. Manimaran (Tharini et al. 2018) conducted a detailed quantitative analysis of the antioxidant activity of ethanol extracts of *F. benghalensis* fruits using multiple assays (Table 3). The DPPH radical scavenging test showed a maximum activity of 75.74% at 60 μg/mL with an IC_50_ of 32.20 μg/mL. In the nitric oxide (NO) scavenging assay, maximum inhibition reached 51.96% ± 3.64% with an IC_50_ of 57.74 μg/mL, while the ABTS assay demonstrated notably high activity (79.57% ± 5.57%) at 30 μg/mL with an IC_50_ of 13.69 μg/mL. Hydroxyl radical scavenging reached 57.02% ± 3.99% (IC_50_ = 34.37 μg/mL). The phosphomolybdenum and ferric reducing assays showed strong total reducing capacities (RC_50_ = 13.78 and 18.71 μg/mL, respectively). Gas chromatography–mass spectrometry (GC–MS) analysis further identified several ester derivatives responsible for these activities, confirming that the fruit extract has broad and potent antioxidant effects through multiple mechanisms.

**TABLE 3:** Summary of antioxidant activity of different extracts and plant parts of *F. benghalensis* .

Plant part	Extract type	Assay method	IC_50_ (μg/mL)	Positive control	References
Fruit	Ethanolic	DPPH	32.20	Ascorbic acid	Amali et al. (2025) and Etratkhah et al. (2019)
Fruit	Ethanolic	ABTS	13.69	Ascorbic acid	Amali et al. (2025)
Fruit	Ethanolic	NO scavenging	57.74	Ascorbic acid	Amali et al. (2025)
Fruit	Ethanolic	Hydroxyl radical scavenging	34.37	Ascorbic acid	Amali et al. (2025)
Stem bark	Methanolic	DPPH	Not reported	Ascorbic acid	Singh, Dhankhar, Kapoor, and Sharma (2023)
Stem bark	Methanolic	ABTS	Not reported	Ascorbic acid	Singh, Dhankhar, Kapoor, and Sharma (2023)
Bark	Hydroalcoholic	DPPH	Not specified	Ascorbic acid	Yadav et al. (2011)
Bark	Hydroalcoholic	ABTS	Not specified	Ascorbic acid	Yadav et al. (2011)
Bark	Hydroalcoholic	HO radical scavenging	Not specified	Ascorbic acid	Yadav et al. (2011)

Etratkhah et al. (2019) focused on the root system, assessing the antioxidant activity of different root extracts of *F. benghalensis* . Phytochemical analysis revealed steroids, flavonoids, tannins, phenolics, and anthraquinone glycosides. Quantitative DPPH and FRAP assays demonstrated antioxidant potential, though the Iranian root extracts exhibited lower activity than Indian ones, reflecting the influence of geographical origin on phytochemical composition. Bhaskara Rao et al. (2014) evaluated methanolic leaf extracts, finding rich contents of carbohydrates, phenolic compounds, flavonoids, alkaloids, saponins, proteins, and tannins. The extracts displayed high antioxidant activity across multiple assays, including DPPH, total antioxidant capacity, iron chelating, and reducing power tests, confirming the leaves as an excellent source of natural antioxidants. Yadav et al. (2011) examined the methanolic latex extract, identifying glycosides, alkaloids, tannins, flavonoids, and amino acids. The total phenolic content was 276.00 mg GAE/kg, and total flavonoids were 1.84 mg QE/kg. The extract exhibited potent antioxidant capacity in several assays, including DPPH (IC_50_ = 28.63 ± 0.16 μg/mL), ferric chloride reducing activity (IC_50_ = 49.82 ± 1.00 μg/mL), and phosphomolybdenum assay (IC_50_ = 31.84 μg/mL), confirming significant radical‐scavenging potential. Bayramli and Arabaci (2025) compared the antioxidant activity of *F. benghalensis* with *Eupatorium maculatum* and *Pinus eldarica* using DPPH and Cupric Reducing Antioxidant Capacity (CUPRAC) assays. The results demonstrated that *F. benghalensis* , with its higher phenolic and flavonoid content, exhibited superior antioxidant capacity among the tested plants, emphasizing its promise for pharmaceutical and functional food applications.

Ramasamy et al. (2022) assessed the antioxidant and acetylcholinesterase (AChE) inhibitory activity of aerial root extracts in solvents of varying polarity. The presence of phenols, alkaloids, flavonoids, glycosides, anthraquinones, tannins, and steroids was confirmed. DPPH and hydroxyl radical scavenging assays revealed that chloroform and ethyl acetate extracts displayed the highest antioxidant activities, which correlated with notable AChE inhibition. This dual action suggests that the antioxidant‐rich medium‐polarity fractions may offer neuroprotective benefits relevant to Alzheimer's disease therapy. Singh et al. (2021) synthesized silver nanoparticles (AgNPs) using *F. benghalensis* extracts and evaluated their antioxidant properties via DPPH assays. A dose‐dependent relationship was observed, with increasing nanoparticle concentration (10–50 μL) enhancing DPPH inhibition up to 90%, demonstrating that nanoformulation preserves and even enhances the antioxidant potential of the plant extract. Pedgaonkar et al. (2019) analyzed methanolic leaf extracts for phenolic content and DPPH activity, reporting total phenolics of 54 μg/100 g extract. The extract exhibited stronger radical scavenging ability than ascorbic acid, establishing a direct positive correlation between phenolic concentration and antioxidant potency.

Althafar (2024) examined the hydroalcoholic bark extract and established a connection between antioxidant and antiproliferative effects on lung cancer A549 cells. The extract demonstrated high antioxidant activity in DPPH and hydrogen peroxide assays and inhibited cell proliferation by 50.12% at 50 μL/mL, indicating potential anticancer and antioxidative synergy. Finally, Gaur et al. (2024) provided a comprehensive review comparing antioxidant capacities among *Ficus* species. The analysis revealed that *F. benghalensis* , *F. carica* , and *F. krishnae* displayed the highest antioxidant activity, with differences influenced by extraction methods, plant parts, and geography. Collectively, these findings underscore *F. benghalensis* as a rich natural reservoir of antioxidants, with low IC_50_ values across radical scavenging assays, high phenolic and flavonoid content, and strong reducing power, highlighting its potential applications in pharmaceuticals, nutraceuticals, and functional foods. The consistent results across diverse extracts and methodologies confirm the plant's value as a source of therapeutic antioxidants with possible neuroprotective and anticancer benefits. Table 3 summarizes the antioxidant activity of different extracts and plant parts of *F. benghalensis* .

*F. benghalensis* shows antioxidant properties through various processes. These are direct phenolic‐compound radical‐scavenging activities, as demonstrated by low IC_50_ values. Metal chelation activity obviates deleterious hydroxyl radical formation (Ramasamy et al. 2022). The extracts exhibit high reducing power in the FRAP assays. The phytochemical profile suggests unconfirmed potential to activate the Nrf2 pathway to upregulate cell protective SOD (Ahmed et al. 2017). The compounds in the plant also inhibit peroxidative membrane damage. Notably, the presence of several synergistically acting antioxidants is combined with high total flavonoids to correlate with markedly high flavonoids (Bhaskara Rao et al. 2014).

### 4.2 Antimicrobial Activity

The antioxidant, antimicrobial, and related bioactive properties of *F. benghalensis* have been consistently supported by quantitative data from multiple studies, highlighting the measurable efficacy of its extracts across different plant parts and experimental models (Table 4). Represent the summary of in vitro antimicrobial studies on *F. benghalensis*. Singh, Dhankhar, Kapoor, and Sharma (2023) reported significant variation in the phytochemical composition of stem bark and aerial roots, with total phenolic, flavonoid, and condensed tannin contents of 75.84 ± 2.70 mg GAE/g, 71.64 ± 3.71 mg QE/g, and 19.25 ± 2.03 mg CE/g, respectively. The same study demonstrated notable antimicrobial activity, showing zones of inhibition (ZOI) of 9.5 ± 0.88 and 6.2 ± 0.88 mm against bacterial strains, and 9.2 ± 1.6 and 6.2 ± 0.90 mm against fungal strains. The minimum inhibitory concentrations (MICs) were remarkably potent, ranging from 50 to 0.024 μg/μL. In a subsequent investigation, Singh, Dhankhar, Kapoor, and Sharma (2023) observed even higher ZOI values for leaf and fruit extracts, with bacterial inhibition zones ranging from 18.8 ± 1.2 to 6.2 ± 0.88 mm, and fungal inhibition zones from 10.2 ± 1.3 to 6.2 ± 1.6 mm, particularly against Gram‐positive organisms. GC–MS analysis further revealed key phytoconstituents such as Lup‐20(29)‐en‐3‐one (20.45%), lupeol (17.40%), octadecatrienoic acid (15.24%), and 5‐hydroxymethylfurfural (15.32%), confirming the dominance of triterpenoids and fatty acids in these extracts (Singh, Dhankhar, Kapoor, and Sharma 2023).

**TABLE 4:** Summary of in vitro antimicrobial studies on *F. benghalensis* .

Plant part	Extract solution	Test organism	Zone of inhibition (ZOI) (mm)	MIC (μg/L)	MBC/MFC value	Positive control	References
Leaves	Methanolic	*Bacillus subtilis*	18.8 ± 1.2 mm	0.024–50	Not reported	Gentamicin	Singh, Dhankhar, Kapoor, and Sharma (2023)
Leaves	Methanolic	*Staphylococcus aureus*	Variable	0.024–50	Not reported	Gentamicin	Singh, Dhankhar, Kapoor, and Sharma (2023)
Leaves	Methanolic	*Salmonella typhi*	Variable	0.024–50	Not reported	Gentamicin	Singh, Dhankhar, Kapoor, and Sharma (2023)
Leaves	Methanolic	*Escherichia coli*	6.2 ± 0.88 mm	0.024–50	Not reported	Gentamicin	Singh, Dhankhar, Kapoor, and Sharma (2023)
Leaves	Methanolic	*Aspergillus niger*	10.2 ± 1.3 mm	0.024–50	Not reported	Fluconazole	Singh, Dhankhar, Kapoor, and Sharma (2023)
Leaves	Methanolic	*Fusarium oxysporum*	Variable	0.024–50	Not reported	Fluconazole	Singh, Dhankhar, Kapoor, and Sharma (2023)
Leaves	Methanolic	*Rhizopus oryzae*	6.2 ± 1.6 mm	0.024–50	Not reported	Fluconazole	Singh, Dhankhar, Kapoor, and Sharma (2023)
Stem bark	Methanolic	*B. subtilis*	9.5 ± 0.88 mm	0.024–50	Not reported	Gentamicin	Singh, Dhankhar, Kapoor, and Sharma (2023)
Stem bark	Methanolic	*S. aureus*	6.2 ± 0.88 mm	0.024–50	Not reported	Gentamicin	Singh, Dhankhar, Kapoor, and Sharma (2023)
Stem bark	Methanolic	*Fungal strains*	9.2 ± 1.6 to 6.2 ± 0.90 mm	0.024–50	MFC: 2× MIC	Fluconazole	Singh, Dhankhar, Kapoor, and Sharma (2023)

Similarly, Tkachenko et al. (2017) described the moderate antimicrobial activity of ethanolic leaf extracts, reporting qualitative sensitivity against selected pathogens, although without numerical ZOI or MIC data. Ogunlowo et al. (2013) compared extracts from various plant parts and identified the stem bark as exhibiting the strongest antimicrobial potential, though results were expressed qualitatively through microbial susceptibility and resistance patterns. Vimala and Shoba (2019), in contrast, reported substantial antimicrobial potency in the ethanolic seed extract, with ZOI values of 19 against *Pseudomonas aeruginosa* , 18 against *Enterococcus faecalis* , and 13 mm against *Aspergillus niger*. High‐Performance Thin Layer Chromatography (HPTLC) profiling revealed phenolic markers such as quercetin (Rf = 0.54) and gallic acid (Rf = 0.45), providing a quantitative basis for these biological effects (Vimala and Shoba 2019).

Abdulhaq and Hershan (2023) examined the *F. benghalensis* methanolic bark extract (FBMB) against *Methicillin‐resistant Staphylococcus aureus* (MRSA) and recorded a MIC of 50 mg/mL and a minimum bactericidal concentration (MBC) of 100 mg/mL. The extract exhibited visible antimicrobial effects even at lower doses of 1.6 mg/mL, producing significant ZOI values (Abdulhaq and Hershan 2023).

In another study, Begum et al. (2025) demonstrated a clear dose‐dependent relationship between the concentration of an *F. benghalensis* ‐based herbal mouthwash and its cytotoxic and antimicrobial activities. In brine shrimp assays, the highest toxicity (80 μL concentration) corresponded with increased mortality, while ZOI values against *Streptococcus mutans* and *Lactobacillus* increased proportionally at concentrations of 25 μg, 50 μg, and 100 μg (Begum et al. 2025). Likewise, Rathod et al. (2024) determined the MICs of various extracts against periodontopathogenic bacteria, reporting values of 1.6 μg/mL for *Aggregatibacter actinomycetemcomitans* and 6.25 μg/mL for *Porphyromonas gingivalis* . A combined alcoholic extract of *F. benghalensis* and *Picrorhiza kurroa* exhibited an MIC of 1.6 μg/mL, indicating synergistic antimicrobial efficacy. Table 3 shows the Summary of in vitro antimicrobial studies on *F. benghalensis* .

Numerous studies claim to observe and report antibacterial properties in various forms; however, upon closer inspection, there is a considerable disparity in the methodology employed. For example, Singh et al. (2021) documented a MIC value range of 50 μg/L to 0.024 μg/L. This represents a 2000‐fold range and can be attributed to the differences in extraction methodologies employed, the sensitivity of the strains of bacteria, and the standardization methodologies, if any, that were used (Singh et al. 2021). Additionally, the absence of studies that investigate the time‐kill kinetics of a given agent restricts our understanding of the mechanisms of action of the component responsible for killing versus the component responsible for inhibition. Comparative analysis of stem bark extracts to the extracts of the leaves and fruits consistently reflects superior activity of the stem bark extracts. This suggests that the distribution of the antimicrobial compounds is not uniform throughout the various parts of the plant.

### 4.3 Antidiabetic Activity

*F. benghalensis* has been widely recognized in traditional medicine for its efficacy in managing diabetes. Recent scientific studies have validated these ethnomedicinal claims, demonstrating their significant effects on blood glucose regulation, insulin secretion, and metabolic enzyme modulation. Modern pharmacological investigations spanning computational, in vitro, ex vivo, and in vivo approaches have elucidated multiple mechanisms underpinning its antidiabetic potential. Table 5 presents the summary of in vitro and in vivo antidiabetic studies on *F. benghalensis*.

**TABLE 5:** Summary of in vitro and in vivo antidiabetic studies on *F. benghalensis* .

Plant part/extract	Experimental model/method	Key findings/results	Proposed mechanism/targets	References
Hydroalcoholic extract (whole tree)	In silico, in vivo (rats)	Reduced blood glucose AUC, increased insulin AUC, and restored hepatic and antioxidant enzymes.	Activation of PI3K/Akt pathway; inhibition of PTP1B; β‐cell protection	Khanal and Patil (2021)
Hydroalcoholic bark extract, fractions	In silico, in vitro, and ex vivo	Strong inhibition of α‐amylase and α‐glucosidase; increased glucose uptake	3,4‐Dihydroxybenzoic acid, ursolic acid, and apigenin as lead inhibitors	Khanal and Patil (2020b)
Hydroalcoholic bark extract	In silico docking, in vitro glucose uptake assay	Maximal glucose uptake at 500 μg/mL; lupeol acetate has strong GLUT‐2 binding (−8.02 kcal/mol)	Enhanced glucose transport via GLUT‐2	Madiwalar et al. (2022)
Leaves, stems, roots (various extracts and fractions)	In vitro enzyme inhibition assays	α‐Glucosidase inhibition (73.84%), α‐Amylase inhibition (76.29%), COX‐2 inhibition (85.72%), and 5‐LOX (87.63%)	Multi‐enzyme inhibition; anti‐inflammatory synergy	Rauf et al. (2024)
Hydroalcoholic extract	Computational	Apigenin, kaempferol, and tetrahydroxyflavones as dual α‐glucosidase and aldose reductase inhibitors	Modulation of p53 and PI3K/Akt pathways	Khanal and Patil (2019)
Whole plant extracts (different solvents)	In vitro enzyme assays	Flavonoid content positively correlated with α‐glucosidase inhibition (*R* = 0.793)	Flavonoids as major active components	Shaikh Abusufyan et al. (2018)
Bark ethanolic extract	In vivo (STZ‐induced diabetic rats)	Dose‐dependent glucose reduction; 500 mg/kg comparable to glibenclamide	Enhanced insulin secretion; glucose homeostasis restoration	Kasireddy et al. (2021)

Abbreviations: 5‐LOX, 5‐Lipoxygenase; AUC, area under curve; COX‐2, Cyclooxygenase‐2; GLUT‐2, glucose transporter 2; PI3K/Akt, phosphoinositide 3‐kinase/protein kinase B pathway; PTP1B, protein tyrosine phosphatase 1B; STZ, streptozotocin.

Khanal and Patil (2021) reported that hydroalcoholic extracts of *F. benghalensis* contain 21 bioactive compounds with predicted interactions against diabetic targets. In silico analysis identified apigenin, 3′,4′,5,7‐tetrahydroxy‐3‐methoxyflavone, and kaempferol as key ligands targeting Protein Tyrosine Phosphatase 1B (PTP1B), while ursolic acid exhibited the strongest binding affinity. Network pharmacology revealed the activation of the PI3K/Akt signaling pathway, correlating with improved glucose tolerance, increased insulin levels, and restoration of hepatic enzyme homeostasis in vivo. The extract enhanced antioxidant enzymes such as catalase and superoxide dismutase, suggesting that its antidiabetic action results from enhanced glycolysis, reduced gluconeogenesis, and protection of pancreatic β‐cells. In another study, Khanal and Patil (2020b) confirmed that hydroalcoholic bark fractions of *F. benghalensis* exhibited strong α‐amylase and α‐glucosidase inhibitory activity, with the flavonoid fraction showing the highest potency. The same extract significantly enhanced glucose uptake in rat hemidiaphragm models and inhibited glucose diffusion. LC–MS analysis identified 3,4‐dihydroxybenzoic acid, ursolic acid, and apigenin as key inhibitors of α‐amylase, PTP1B, and α‐glucosidase, respectively, collectively responsible for the extract's hypoglycemic effects. Madiwalar et al. (2022) combined computational and experimental approaches to identify glucose uptake enhancers. Among 17 screened phytoconstituents, 4‐methoxybenzoic acid exhibited the highest drug‐likeness score, while lupeol acetate displayed the strongest binding affinity (−8.02 kcal/mol) with the GLUT‐2 transporter. In vitro assays with baker's yeast confirmed maximal glucose uptake at 500 μg/mL of hydroalcoholic bark extract.

Rauf et al. (2024) evaluated extracts from leaves, stems, and roots, showing high inhibitory activity against α‐glucosidase (73.84%) and α‐amylase (76.29%) at 1000 μg/mL. The leaf and stem extracts, along with column fractions (F‐B‐2C and F‐B‐3C), also demonstrated anti‐cholinesterase and anti‐inflammatory properties, with COX‐2 inhibition of 85.72% and 5‐LOX inhibition of 87.63%. Another computational study by Khanal and Patil (2019) identified apigenin, kaempferol, and 3,4′,5,7‐tetrahydroxy‐3,7‐methoxyflavone as dual inhibitors of α‐glucosidase and aldose reductase, indicating that *F. benghalensis* exerts multi‐targeted effects via the p53 and PI3K/Akt pathways. Murugesu et al. (2021) reviewed the broad pharmacological properties of *F. benghalensis* , highlighting its rich phytochemical diversity and well‐documented antidiabetic and antioxidant activities. In a comparative analysis, Shaikh Abusufyan et al. (2018) found a strong correlation (*R* = 0.793) between total flavonoid content and α‐glucosidase inhibition, emphasizing flavonoids as primary antidiabetic agents.

In vivo validation by Kasireddy et al. (2021) demonstrated a dose‐dependent antihyperglycemic effect in streptozotocin‐induced diabetic rats. Oral administration of ethanolic bark extract (150, 300, and 500 mg/kg) for 15 days significantly reduced fasting blood glucose levels. The 500 mg/kg dose exhibited activity comparable to the standard antidiabetic drug glibenclamide (0.5 mg/kg). Collectively, these studies provide robust mechanistic and quantitative evidence that *F. benghalensis* exerts its antidiabetic effects through enzyme inhibition, enhanced glucose uptake, insulin sensitization, and antioxidant protection.

Although Khanal et al.'s network pharmacology and docking studies determined apigenin and ursolic acid to be key bioactives, there are some differences between the computational binding affinities and their experimental counterpart. Despite the computational model predicting strong binding of GLUT‐2 to lupeol acetate (−8.02 kcal/mol), in vitro glucose uptake assays indicated maximal effects at 500 μg/mL, which may suggest moderate bioavailability or some limitations in cellular uptake.

### 4.4 Anti‐Inflammatory Potential

*F. benghalensis* exhibits strong anti‐inflammatory potential that has been verified through both in vitro and in vivo investigations (Table 6). Its extracts and isolated bioactive compounds demonstrate diverse molecular mechanisms that validate its traditional use in treating inflammatory and rheumatic disorders (Rauf et al. 2024; Halyna et al. 2017; Imran et al. 2021; Baburaj et al. 2022; Deore et al. 2012). Rania Alaaeldin et al. (2022) examined the anti‐inflammatory activity of a Fatty Acid Glucoside (FAG) derived from *F. benghalensis* in lipopolysaccharide (LPS)‐induced RAW 264.7 macrophages. When applied at a non‐cytotoxic concentration, FAG stimulated nitric oxide (NO) release and enhanced the activity of inducible nitric oxide synthase (iNOS), while inhibiting the enzymes cyclooxygenase‐1 (COX‐1) and cyclooxygenase‐2 (COX‐2), leading to reduced prostaglandin E2 (PGE2) levels. At both genetic and protein expression levels, FAG downregulated the pro‐inflammatory cytokines interleukin‐6 (IL‐6) and IL‐1β, as well as the epidermal growth factor receptor (EGFR), Akt, and PI3K proteins. Molecular docking analysis indicated that FAG exhibited high‐affinity binding to EGFR, suggesting that it acts as a natural EGFR inhibitor and modulator of the EGFR/Akt/PI3K signaling pathway while simultaneously promoting NO release and COX inhibition.

**TABLE 6:** Summary of in vitro and in vivo anti‐inflammatory studies on *F. benghalensis* .

Extract/Compound	Model/System	Dose/Concentration	Key findings/% inhibition	Mechanism	References
Fatty acid glucoside (FAG)	LPS‐induced RAW 264.7 macrophages	Non‐cytotoxic dose	↑NO, ↑iNOS, ↓COX‐1/2, ↓PGE2; ↓IL‐6, ↓IL‐1β	EGFR/Akt/PI3K inhibition; high EGFR binding affinity	Alaaeldin et al. (2022)
α‐Amyrin acetate, stigmasterol acetate	Carrageenan‐induced rat paw edema	100–200 mg/kg	42.5% (stigmasterol), 45% (α‐amyrin acetate), Indomethacin 62.5%	COX inhibition: dose‐dependent effect	Mazumder et al. (2022)
α‐Amyrin (Fb vs. Ab)	In vitro assays (albumin denaturation, anti‐proteinase, anti‐lipoxygenase)	Not specified	Comparable effect; Fb yields higher (0.42 mg vs. 0.23 mg)	α‐Amyrin inhibits proteinase/lipoxygenase; Fb superior source	Baburaj et al. (2022)
Aqueous, chloroform, and methanolic bark extracts	HRBC membrane stabilization	200 mg/mL	Significantly, the methanolic extract most active	Stabilizes membranes; comparable to diclofenac sodium	Matpal et al. (2013)
Methanolic leaf extract	Carrageenan‐induced paw edema; acetic acid writhing test	10–100 mg/kg	Significant inhibition (*p* < 0.05); analgesic and anti‐inflammatory	COX inhibition; reduced edema and writhing	Mahajan et al. (2012)
Methanolic leaf extract	Formalin‐induced paw edema	200 mg/kg	65.21% inhibition vs. Diclofenac 62.31%	Potent anti‐inflammatory and analgesic action	Kothapalli et al. (2014)
Aqueous aerial root extract	Carrageenan and cotton pellet‐induced inflammation	100–200 mg/kg	Dose‐dependent inhibition	Effective in acute and chronic inflammation models	Deore et al. (2012)
Review of phytochemicals	Literature synthesis		Summarized terpenoids, flavonoids, and sterols	Calls for further in vivo and clinical validation	Tahir et al. (2023)
CuO nanoparticles were synthesized using the extract	In vitro	10–50 μL	11%–92.7% inhibition (dose‐dependent)	Enhanced anti‐inflammatory efficacy via nanoformulation	Nandita and Rajeshkumar (2023)

Further evidence of *F. benghalensis* 's anti‐inflammatory potential comes from high‐performance liquid chromatography (HPLC) purification and characterization of two bioactive acetylated compounds, 1,25‐α‐amyrin acetate and stigmasterol acetate, from methanolic extracts of aerial roots (Mazumder et al. 2022). When tested in rats using the carrageenan‐induced paw edema model, the ethyl acetate extract at 200 mg/kg inhibited inflammation by 25% and 47.5%, respectively. At a dose of 100 mg/kg, stigmasterol acetate and α‐amyrin acetate inhibited paw edema by 42.5% and 45%, compared with the standard drug indomethacin, which showed 62.5% inhibition. These results confirmed that the compounds possessed potent, dose‐dependent anti‐inflammatory properties. Baburaj et al. (2022) compared α‐amyrin isolated from *F. benghalensis* (Fb) stem bark and *Alstonia boonei* (Ab). The Fb source produced a higher yield (0.42 mg) than the Ab source (0.23 mg). The α‐amyrin compound demonstrated comparable, dose‐dependent in vitro anti‐inflammatory activity in albumin denaturation, anti‐proteinase, and anti‐lipoxygenase assays for both species, though *F. benghalensis* was identified as a superior source due to its higher yield.

Matpal et al. (2013) investigated the anti‐inflammatory potential of aqueous, chloroform, and methanolic bark extracts using the human red blood cell (HRBC) membrane stabilization assay, finding that all extracts exhibited significant activity. The methanolic extract at a concentration of 200 mg/mL showed the strongest membrane‐stabilizing effect, comparable to that of diclofenac sodium. Mahajan et al. (2012) reported that oral administration of the methanolic leaf extract at doses of 10, 20, and 100 mg/kg body weight produced significant (*p* < 0.05) anti‐inflammatory effects by reducing carrageenan‐induced hind paw edema in rats. The extract also demonstrated notable analgesic effects by reducing acetic acid‐induced writhing and prolonging reaction time in Eddy's hot plate method, confirming both anti‐inflammatory and analgesic properties.

Similarly, Kothapalli et al. (2014) found that methanolic leaf extract at a dose of 200 mg/kg significantly (*p* < 0.001) reduced paw edema volume by 65.21% in a formalin‐induced inflammation model, closely comparable to diclofenac, which inhibited 62.31%. The extract also produced a significant analgesic effect in the hot plate assay, supporting its dual activity. Deore et al. (2012) demonstrated that aqueous aerial root extracts at doses of 100 and 200 mg/kg produced a dose‐dependent reduction in inflammation in carrageenan‐induced paw edema and cotton pellet‐induced granuloma models, confirming the anti‐inflammatory efficacy of this plant part and extraction method. Tahir et al. (2023) synthesized findings from existing literature, noting that the anti‐inflammatory potential of *F. benghalensis* is associated with its diverse phytochemicals, including terpenoids, flavonoids, and sterols, while emphasizing the need for further in vivo and clinical studies. Nandita and Rajeshkumar (2023) explored the synthesis of copper oxide nanoparticles using *F. benghalensis* extract, reporting concentration‐dependent anti‐inflammatory effects across tested volumes (10–50 μL), with inhibition rates ranging from 11% to 92.7%. This study highlighted the remarkable enhancement of anti‐inflammatory potential achieved through nanoformulation. Another study involved the fractionation of aerial root extract of *F. benghalensis* to isolate bioactive constituents (such as α‐amyrin acetate and stigmasterol), which were evaluated for anti‐inflammatory activity in rats using the carrageenan‐induced paw oedema model. The reduction of inflammation was significant at 200–400 mg/kg (extract) and 100 mg/kg (isolated compounds) with inhibition of paw volume 25% (200 mg/kg extract), 47.5% (400 mg/kg extract), 42.5% (stigmasterol), and 45% (α‐amyrin acetate) after 3 h compared with indomethacin (standard drug) 62.5%. The results showed that the aerial roots of *F. benghalensis* have significant anti‐inflammatory activity primarily due to its active components, α‐amyrin acetate and stigmasterol (Mazumder et al. 2022).

Collectively, available evidence demonstrates that *F. benghalensis* possesses strong anti‐inflammatory potential supported by consistent in vitro, ex vivo, and in vivo findings. Studies report substantial quantitative effects, with inhibition rates ranging from 25% to 92.7%, alongside modulation of key inflammatory mediators, enzymes, and signaling pathways (Rauf et al. 2024). These effects are largely attributed to bioactive constituents such as fatty acid glucoside, which inhibits COX‐1/COX‐2 and prostaglandin E2 production, suppresses EGFR‐mediated PI3K/Akt and NF‐κB signaling, and reduces pro‐inflammatory cytokines, while other extracts contribute through membrane stabilization, 5‐lipoxygenase inhibition, and antioxidant defense (Alaaeldin et al. 2022). The reproducibility of these outcomes across different plant parts and experimental models highlights *F. benghalensis* as a promising natural anti‐inflammatory agent; however, the absence of randomized controlled clinical trials represents a critical knowledge gap, emphasizing the need for well‐designed human studies to validate its therapeutic potential (Mazumder et al. 2022).

### 4.5 Anticancer Activity

*F. benghalensis* has emerged as a promising lead for anticancer drug development, exhibiting potent cytotoxic, antiproliferative, and proapoptotic activities across multiple cancer cell lines (Hassan et al. 2022; Cabanlit et al. 2025; Khanal and Patil 2020a; Lansky et al. 2008). Its efficacy is largely attributed to a rich profile of phytochemicals, including flavonoids, phenolics, triterpenoids, and novel bioactive molecules. Dutta et al. (2022) investigated the anticancer activity of methanolic leaf extracts from *F. benghalensis* and other *Ficus* species against breast cancer cells. The extract exhibited selective cytotoxicity, significantly reducing breast cancer cell viability at concentrations as low as 5 μg/mL, while sparing normal breast cells even at higher doses. This finding demonstrates a strong therapeutic window and highlights the potential of *F. benghalensis* as a selective anticancer nutraceutical or chemopreventive agent. Althafar (2024) evaluated the antiproliferative potential of hydroalcoholic bark extracts on human lung carcinoma (A549) cells. The extract inhibited 50.12% of cell proliferation at a concentration of 50 μg/mL, corresponding to the IC_50_ value. Morphological and viability assays confirmed significant cellular deformation and death among treated cells (Dutta et al. 2022). Mechanistic assays revealed increased oxidative stress, evidenced by nitric oxide release and lipid peroxidation, alongside the upregulation of apoptosis‐related genes such as *Bax*, *c‐MYC*, and *PARP*. These findings demonstrate that *F. benghalensis* bark extract exerts its anticancer effects through oxidative stress induction and activation of apoptotic pathways (Althafar 2024).

Hassan et al. (2022) isolated three bioactive compounds from *F. benghalensis* leaves and tested them against the HL60 leukemia cell line. Among these, carpachromene displayed the strongest antiproliferative effect, inducing apoptosis and causing cell cycle arrest at the G_2_/M phase. Molecular docking and western blot analyses confirmed that carpachromene interacts with and inhibits topoisomerase I, leading to downregulation of its protein expression and upregulation of pro‐apoptotic genes. This study provides clear mechanistic evidence linking carpachromene to cell cycle regulation and apoptosis induction, identifying it as a key anticancer agent in *F. benghalensis* leaves. Tulasi, Lakshmi, and Saida (2018) conducted an extensive screening of solvent extracts of *F. benghalensis* latex for cytotoxicity against breast (MDA‐MB‐231), colorectal (HCT116), and neuroblastoma (IMR‐32) human cancer cell lines. The ethanol extract exhibited the strongest inhibition against colorectal (HCT116) and neuroblastoma (IMR‐32) cells with IC_50_ values of 99.82 μg/mL and 123.27 ± 2.5 μg/mL, respectively, whereas the ethyl acetate extract was most active against the breast cancer line (MDA‐MB‐231) with an IC_50_ of 75.66 ± 6.3 μg/mL. Importantly, the extracts demonstrated reduced toxicity toward peripheral blood lymphocytes, confirming selective cytotoxicity. These results suggest that the latex possesses broad‐spectrum anticancer properties, varying with solvent polarity and cancer type.

Nayak et al. (2016) advanced the field through the green synthesis of silver nanoparticles (AgNPs) using *F. benghalensis* bark extract. These AgNPs displayed dose‐dependent antiproliferative activity against osteosarcoma (MG‐63) cells, where increased nanoparticle concentrations correlated with higher cancer cell inhibition. The study emphasized the potential of nanostructured derivatives of *F. benghalensis* as efficient bio‐nanomedicine candidates for bone cancer therapy (Nayak et al. 2016). Gurav et al. (2022) further explored nanotechnology applications by utilizing leaf extracts of *F. benghalensis* for the synthesis of Fe_3_O_4_ magnetic nanoparticles, later functionalized into a novel Fe_3_O_4_@Ag‐S‐CH_2_‐COOH nanocatalyst. This system was applied for the synthesis of 3,4‐dihydropyrimidin‐2 (1H)‐one derivatives, which exhibited cytotoxic activity against Hep‐G2 liver cancer cells. Anti‐angiogenic and molecular docking assays supported these results, highlighting that *F. benghalensis* ‐derived nanocomposites can serve as platforms for producing novel anticancer compounds. Collectively, these findings establish *F. benghalensis* as a robust, multi‐mechanistic anticancer source. Its bioactive extracts exhibit potent cytotoxicity at low micromolar concentrations (as low as 5 μg/mL), selective killing of cancer cells over normal cells, and diverse mechanisms including oxidative stress induction, apoptosis, topoisomerase inhibition, and G_2_/M cell cycle arrest. Furthermore, the integration of *F. benghalensis* phytochemicals into nanoparticle systems demonstrates enhanced efficacy and specificity, making this species a valuable candidate for developing next‐generation anticancer therapeutics and drug delivery platforms. Table 7 shows the summary of anticancer activity of *F. benghalensis* extracts and isolated compounds against various human cancer cell lines.

**TABLE 7:** Quantitative anticancer/cytotoxic activity data for *F. benghalensis* .

Plant part/extract	Cancer cell line	IC_50_ (μg/mL)	Selectivity index (SI)	Normal cell IC_50_	Key mechanism(s) of action	Positive control	References
Leaf (methanolic extract)	Breast cancer cells	5	> 2.6	Unaffected at higher doses	Selective cytotoxicity; potential chemopreventive activity; induction of apoptosis	Doxorubicin	Murugesu et al. (2021), Mazumder et al. (2022), Gurav et al. (2022), and Tulasi, Narasu, and Saida (2018)
Bark (hydroalcoholic extract)	Lung (A549)	50.12	—	—	Induces oxidative stress, lipid peroxidation, apoptosis via ↑Bax, ↑c‐MYC, ↑PARP; enhanced antioxidant activity	Doxorubicin	Tahir et al. (2023) and Cherian and Augusti (1993)
Leaf (isolated compounds—Carpachromene)	Leukemia (HL60)	Most active (G_2_/M arrest)	—	—	Topoisomerase I inhibition (docking score: −8.5 kcal/mol); G_2_/M phase cell cycle arrest; apoptosis induction via ↑cleaved caspase‐3, ↑Bax, ↑PARP	Topoisomerase I inhibitors (clinical standard)	Mahajan et al. (2012)
Latex (ethyl acetate extract)	Breast (MDA‐MB‐231)	75.66 ± 6.3	> 2.6	PBL: > 200 μg/mL	Selective cytotoxicity to cancer cells; minimal toxicity to peripheral blood lymphocytes (PBL)	Doxorubicin	Mazumder et al. (2022), Cherian and Augusti (1993), and Kothapalli et al. (2014)
Latex (ethyl acetate extract)	Colorectal (HCT116)	99.82	Not calculated	PBL: > 200 μg/mL	Selective cytotoxicity to cancer cells; minimal toxicity to peripheral blood lymphocytes (PBL)	Doxorubicin	Tahir et al. (2023) and Dutta et al. (2022)
Latex (ethanol extract)	Neuroblastoma (IMR‐32)	123.27 ± 2.5	Not calculated	PBL: > 200 μg/mL	Selective cytotoxicity to cancer cells; minimal toxicity to peripheral blood lymphocytes (PBL)	Doxorubicin	Mazumder et al. (2022) and Tulasi, Lakshmi, and Saida (2018)
Bark‐derived silver nanoparticles (AgNPs)	Osteosarcoma (MG‐63)	Dose‐dependent	—	—	Enhanced antiproliferative activity via nanoparticle formulation; dose‐dependent inhibition of cancer cell proliferation	Standard nanoparticle therapy	Khanal and Patil (2020a)
Leaf extract–derived Fe_3_O_4_@Ag nanocatalyst	Liver (Hep‐G2)	Potent (anti‐angiogenic)	—	—	Green nanocomposite synthesis; anti‐angiogenic effects; catalytic potential for novel anticancer compound synthesis	Anti‐angiogenic agents	Lansky et al. (2008)

Critically assessing selectivity is one of the cornerstones of anticancer drug discovery. Tulasi, Narasu, and Saida (2018) documented that the ethyl acetate latex extract had an IC_50_ of 75.66 ± 6.3 μg/mL against MDA‐MB‐231 breast cancer cells and significantly lower toxicity against peripheral blood lymphocytes (IC_50_ > 200 μg/mL), resulting in an SI of > 2.6. Colorectal (HCT116) and neuroblastoma (IMR‐32) cell lines recorded IC_50_ values of 99.82 and 123.27 ± 2.5 μg/mL, respectively, with minimal toxicity to normal cells, demonstrating favorable therapeutic windows, or as we have defined it, selectivity (Tulasi, Narasu, and Saida 2018).

### 4.6 Comparison With Standard Chemotherapeutics: Enhanced Efficacy and Multi‐Targeted Mechanisms

The anticancer effectiveness of *F. benghalensis* extracts and isolated compounds developed is even more pronounced when conducted in a systematic comparison with standard pharmaceutical drugs currently being used in clinical practice. Although doxorubicin, which is one of the most commonly used topoisomerase II inhibitors, displays IC_50_ 0.5–2 μg/mL at different cancer cell lines (Tahir et al. 2023), *F. benghalensis* extracts are found to have a range of IC_50_ between 5 and 125 μg/mL. This apparently broader IC_50_ span and greater values portray not decreased therapeutic capability, but different drug pharmacological character and mechanistic benefits. The comparison of the lower apparent potency of isolates in cell cultures might be balanced with the multi‐targeted treatment in plant‐derived compounds, which can work in many different cellular signaling streamlines simultaneously (Yakkala et al. 2023).

One of the key differences between synthetic and natural products is their cellular selectivity of cytotoxicity and collateral damage. Commonly used chemotherapeutic drugs such as doxorubicin obtain their anticancer effects by non‐selective DNA intercalation, topoisomerase poisoning, which inevitably affects normal cellular functionality and causes severe systemic toxicity (Yakkala et al. 2023). Conversely, extracted *F. benghalensis* and phytochemicals show lower levels of negative cytotoxicity but still possess multi‐targeted mechanisms (Tahir et al. 2023). Such a phenotype predisposes plant‐derived agents as some of the most attractive with respect to combination therapy strategies and applications of chemoprevention methods where preventing excessive harm to normal tissues but focusing on tumor cells is the most significant property (Murugesu et al. 2021). The most notable isoprenoid compound and one representative of this therapeutic benefit is carpachromene, a compound of *F. benghalensis* leaves. Similar to some clinical topoisomerase I inhibitors such as irinotecan and topotecan, carpachromene has adequate structure and functionality to support full structural optimization and medicinal development (Ramalingam et al. 2023). This duality in which the compound rapidly and specifically inhibits topoisomerase I and at the same time triggers oxidative stress response and mitochondrial machinery associated with apoptosis makes it superior to traditional monotherapy chemotherapeutics (Song et al. 2021). The carpachromene docking of −8.5 kcal/mol against topoisomerase I is similar to that obtained with approved enzyme topoisomerase inhibitors, which implies a similar association with the target enzyme (Eldehna et al. 2026).

Also, some new evidence suggests that combination specific channels using *F. benghalensis‐*derived compounds with conventional chemotherapeutics can deliver better therapeutic outcomes as opposed to monotherapies. Natural products have shown tremendous ability to help sensitize chemoresistant cancer cells to conventional ones but also decrease the toxicity associated with chemotherapy (Talib et al. 2024). Various sources reported the potentiation of traditional chemotherapeutic agents by such plant polyphenols as quercetin and kaempferol, which were found in tissues of *F. benghalensis* (related to the modulation of oxidative stress and the potentiation of the apoptotic pathway) (Jomova et al. 2025). The observed benefit of plant extracts is therefore due to their ability to tune redox homeostasis and activate several survival pathways concomitantly, which is a mechanism that bypasses the developed resistance mechanism with single‐agent chemotherapy (Talib et al. 2021). *F. benghalensis* ‐produced compounds have a pharmacokinetic profile, which is also worth considering in comparison with synthetic drugs. Numerous natural products reveal good bioavailability, less systemic accumulation in non‐target tissues, and metabolic transformation to bioactive derivatives inside target cancer tissues (Stephen et al. 2025). These characteristics assist in enhancing a therapeutic window, the range between the therapeutic and toxic doses, which is often broader than that of standard chemotherapeutics (Gok et al. 2023). Moreover, the chemical complexity of plant extracts, although posing analytical limitations, offers their own benefits in terms of preventing the emergence of acquired chemoresistance, since a cancer cell has to regulate various corrective mechanisms in response to multi‐targeted botanical therapies (Li, Yang, et al. 2024).

### 4.7 Molecular Targets and Mechanistic Insights: DNA Damage, Cell Cycle Arrest, and Apoptotic Cascades

Mechanistic studies into *F. benghalensis* ‐induced anticancer effects have identified complex molecular networks which control cancer cell death with specific focus on topoisomerase inhibition, induction of DNA damage and multi‐pathway apoptotic networks. Hassan et al. showed that carpachromene, with a direct binding specificity to topoisomerase I (docking score: −8.5 kcal/mol), causes disastrous DNA damage which includes strand breaks, a loss of secondary structure, and eventual cell arrest in the G2/M phase in HL60 acute promyelocytic leukemia cells (Yakkala et al. 2023; Hassan et al. 2014). Western blot banding of treated cells showed significant downregulation of topoisomerase I protein, accompanied by concomitant upregulation of classical indicators of apoptosis such as cleaved caspase‐3, pro‐apoptotic protein Bax and fragmented PARP (poly‐ADP‐ribose polymerase), demonstrating strong induction of the intrinsic mitochondrial death pathway (Eldehna et al. 2026). Mechanistic evidence of direct causality between topoisomerase targeting and tumor cell elimination has been demonstrated by the temporal sequence of molecular events, which include: topoisomerase I inhibition before the cell cycle checkpoint is triggered, and apoptotic commitment (Ahmed et al. 2021).

G2/M arrest caused by carpachromene and carpachromepenilike compounds from *F. benghalensis* demonstrates a disturbance of the mechanobiological control systems necessary to mediate an unimpeded progression into mitosis after assurance of full and accurate replication of the DNA (Yadav et al. 2024). Particularly, *F. benghalensis* extract treatments routinely lower expressions of the following elements of transit through the G2/M checkpoint: cyclin‐dependent kinase 1 (CDK1), cyclin B1, and cdc25c phosphatase (Wang et al. 2018). Simultaneously increased phosphorylated checkpoint kinase 1 (p‐CHK1) and 2 (p‐CHK2) and accumulation of phosphorylated histone H2AX (γ‐H2AX) as a surveillance of DNA double‐strand breaks, respectively, confirm the successful retrieval of the DNA damage response (DDR) machinery. This artificial recap is a combination of checkpoint‐activating and prolonged CDK inhibition reaction that successfully traps cancer cells in G2 phase, inhibiting aberrant mitosis and permitting DNA repair or allowing irreversible dedication to apoptosis (Chen et al. 2024).

Afşin et al. (2026) showed that *F. benghalensis* bark extracts substantially cause oxidative stress in A549 lung adenocarcinoma cells by many mechanisms, including increasing the synthesis of nitric oxide (NO) and elevating biomarkers of lipid peroxidation, as well as decreasing antioxidant cellular defenses. Mechanistically connected with these oxidative perturbations are pronounced overexpression of oncogenic c‐MYC transcription factor and pro‐apoptotic Bax protein, which are associated with ongoing activation of intrinsic mitochondrial apoptotic cascades (Yameny and Fekry 2026). Apoptosis of these cells is mediated by oxidative stress, which includes a prolonged increase in mitochondrial reactive oxygen species (ROS), the depletion of the mitochondrial membrane potential, the release of cytochrome c into the cytoplasm, and the formation of an apoptosome complex (consisting of cytochrome c, apoptotic protease‐activating factor‐1, and pro‐casp). The products of lipid peroxidation, especially malondialdehyde (MDA) and 4‐hydroxynonenal (HNE), accumulate as a part of this process and play a direct role in apoptotic signaling via various mechanisms such as membrane integrity disruption, anti‐apoptotic protein inactivation through posttranslational modification, and pro‐death signaling pathway activations. *F. benghalensis* phytochemicals not only activate intrinsic mitochondrial‐dependent mechanisms of multi‐targeted apoptotic pathways, but also extrinsic death receptor pathways (Hassan et al. 2014). A few pieces of evidence suggest that a compound of *F. benghalensis* can upregulate the expression of death receptors (Fas/FasL) on the surface of cancer cells, the recruitment and activation of the death‐inducing signaling complex (DISC), and, ultimately, the activation of caspase‐8 initiator protease (Ting‐Wen et al. 2017). This initiator caspase then cleaves and activates executioner caspase‐3 directly via the extrinsic pathway or indirectly via executioner caspase‐3‐activating amplification loops involving the cleavage of BID and the mitochondrial outer membrane permeabilization (Pal et al. 2021). The overlap of two extrinsic and intrinsic apoptotic pathways activated by single compounds of *F. benghalensis* offers redundant death signals that overwhelm counter‐mechanisms against apoptosis and leave malignant cells extremely vulnerable to treatments (Liao et al. 2023).

Moreover, new mechanistic evidence attributes endoplasmic reticulum (ER) stress pathways to *F. benghalensis* ‐induced apoptosis, especially when cancer cells are adjusted to the situation of high basal oxidative and proteotoxic stress (Liao et al. 2023). The inositol‐requiring enzyme‐1 alpha (IRE1alpha) and PKR‐like endoplasmic reticulum kinase (PERK) arms of the unfolded protein response (UPR) are overwhelmingly activated in response to the DNA fracture and oxidative strain throughout topoisomerase inhibition (Wei et al. 2021). Prolonged UPR activation gradually overwhelms the protein‐folding power of the cell, enhances the formation of stress‐granule complexes of pro‐apoptotic components, and ultimately redirects cellular metabolism to energy loss and pro‐apoptotic commitment (Chuang et al. 2025). Such ER stress‐mediated apoptosis regularly involves pro‐apoptotic BH3‐only proteins (BIM, NOXA, and PUMA) via processes attentive to transcription factor C/EBP‐homologous protein (CHOP) inductions, which interact with mitochondrial‐mediated apoptosis to guarantee irreversible cell death in cancer cells (Talib et al. 2024).

## 5 Toxicity, Safety, and Pharmacokinetics

*F. benghalensis* has been studied extensively, but only a few spaced‐out studies have been conducted concerning its toxicity and pharmacokinetics. Safety studies show that the aqueous and ethanolic extracts were well tolerated by the test subjects at the therapeutic dose (Salehi et al. 2021). Amali et al. (2025) studied the blood parameters of female albino rats and found that ethanolic leaf and bark extracts did not have any significant effects on the red blood cells, the hemoglobin, or the white blood cells, even at high doses of 500 mg/kg. However, concentrated latex and bark extracts can cause mild gastrointestinal and skin hypersensitivity, which makes careful dose optimization necessary (Amali et al. 2025).

There have been no pharmacokinetic studies. β‐sitosterol, lupeol, and apigenin have been shown to have efficacy both in vitro and in vivo, but bioavailability, plasma half‐life, tissue distribution, and metabolic pathways have all been largely left out of studies. The lipophilic nature of the major terpenoids suggests a lack of bioavailability, which could be solved by the proposed methods of nanoencapsulation, liposomal delivery, and the use of bioenhancers (Sasidharan et al. 2023).

Standardization of crude extracts is being complicated by the seasonal and geographical differences in the composition of phytochemicals, and extracts that contain bioactives that are poorly soluble in water, which necessitates the use of a solubilization strategy, some phenolic compounds that are chemically unstable during extraction and storage, and the absence of analytical markers that are uncontested are all formulation challenges. Future standardized phytopharmaceuticals will need the attainment of Good Manufacturing Practices (GMP), HPLC‐validated fingerprinting, and studies on shelf‐life stability (Ramasamy and Kathiresan 2023).

## 6 Comparative Analysis With Other Ficus Species

**FIGURE 3:**
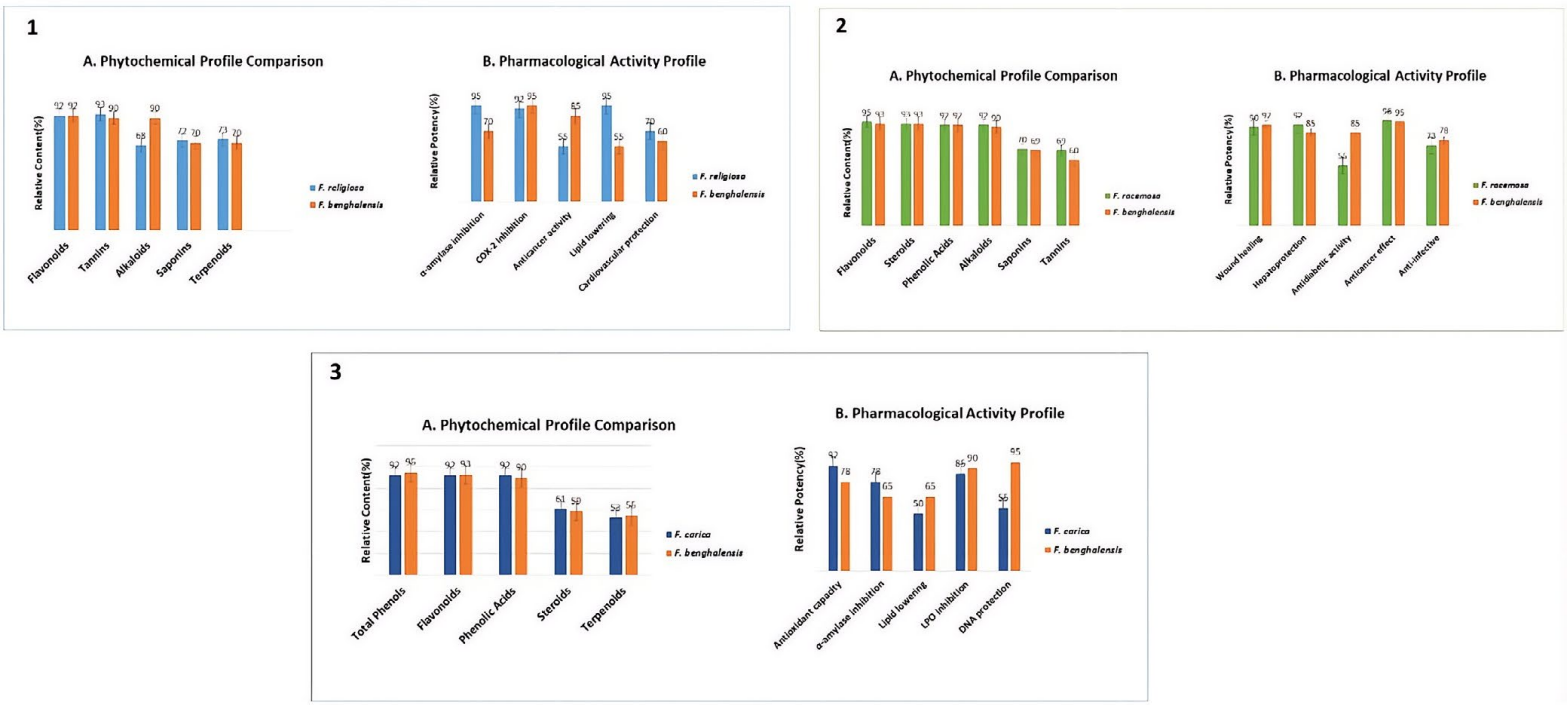
Comparative analysis of phytochemical composition and pharmacological activity profiles among selected *Ficus* species. Box 1 illustrates the phytochemical profile comparison (A) and pharmacological activity profile comparison (B) between *F. religiosa* and *F. benghalensis* , highlighting shared phytochemical classes and variations in biological activities. Box 2 presents the phytochemical profile comparison (A) and pharmacological activity profile comparison (B) between *F. racemosa* and *F. benghalensis* , demonstrating similarities in phytochemical composition alongside differences in therapeutic specialization. Box 3 depicts the phytochemical profile comparison (A) and pharmacological activity profile comparison (B) between *F. carica* and *F. benghalensis* , emphasizing comparable phytochemical abundance with divergent pharmacological performance and research status.

The genus *Ficus* comprises over 800 species, hence the similarity in the traditional perspective of their medicinal uses. However, a recent study (Madrigal‐Santillán et al. 2024) demonstrates that their phytochemical and pharmacological profiles are quite different. A comparison of the chemical and therapeutic profiles of four species—*F. benghalensis*, *F. religiosa*, *F. racemosa*, and *F. carica* was carried out in Figure 3. It proves that despite the similarity of their bioactive compositions, their pharmacological efficacy is different (Gaur et al. 2024). Such variations enable us to give emphasis on a particular species that we develop to treat instead of treating all *Ficus* species homogenously.

### 6.1 *F. religiosa*

As shown in Figure 3, the phytochemical profiles of the plant species are nearly equal: flavonoids (92% vs. 92%), tannins (97% vs. 99%), and terpenoids (73% vs. 70%). This is due to a similarity in the biochemical foundations underlying the antioxidant and antidiabetic properties of both species (Murugesu et al. 2021). The alkaloid content has some minor variations, but in a way that there is higher content in *F. benghalensis* (90%) than in *F. religiosa* (80%), which could be the reason behind its higher anti‐inflammatory and anticancer effects (Arya et al. 2025). Further functional differences are indicated by pharmacological tests. The lipid‐lowering effect of *F. religiosa* is larger (95% vs. 55%), and it has been more widely researched in cardiovascular protection. However, in comparison, the COX‐2 (95% vs. 92%) and anticancer (85% vs. 58%) inhibition is better in *F. benghalensis* (Abu‐Izneid et al. 2024), suggesting a more effective potential on both inflammation‐mediated and selective antiproliferative processes (Singh, Dhankhar, Kapoor, et al. 2023). Therefore, the therapeutic effects are largely different, though phytochemical concentrations are very close, and it is necessary to consider the biological activity of a specific medication and not only rely on the presence of phytochemicals.

### 6.2 *F. racemosa*

The comparison between *F. racemosa* and *F. benghalensis* has demonstrated that there is a great overlap in phytochemicals, whereas distinct differences exist in the pharmacological applications (Hasan et al. 2024). Figure 3 reveals that the two species have approximately 97% flavonoids, steroids, and phenolic acids, and this justifies why they are widely used in diabetes and infection control. Saponins are varied, and *F. racemosa* is 69% as compared to *F. benghalensis* , 60%. *F. racemosa* has been found to heal wounds more effectively (92%) and protect the liver (90%) compared to *F. benghalensis* (both 85%), and is consistent with its usage in tissue repair and liver disease (Roohi et al. 2025). At the same time, *F. benghalensis* is more effective in antidiabetic effect (85% vs. 56%) and agreeable to *F. racemosa* in anti‐inflammatory efficacy (both 95%), meaning it shows a greater impact on the metabolic state and inflammation. Therefore, even with many similarities in terms of phytochemicals that are found in both plants, they are stronger in different medical applications.

### 6.3 *F. carica*

The analysis of the total phenolic and flavonoid contents of *F. carica* and *F. benghalensis* shows that the phenols and flavonoid content are nearly similar (92%–95% and 92%–93%, respectively) (Figure 3), qualifying their similar antioxidant activity and antiproliferative power (Boukhalfa et al. 2019). The levels of alkaloids are also quite similar (92% in *F. carica* as compared to 90% in *F. benghalensis* ), and this biochemical similarity strengthens this comparison. However, pharmacological profiling indicates some significant differences in functional outcomes. *F. carica* has better antidiabetic activity (95%) than *F. benghalensis* (78%), with greater clinical and experimental evidence of its application in the regulation of glycemic control. In its turn, *F. benghalensis* demonstrates superior cytoprotective activity (85% vs. 65%) with better lipid‐lowering (85% vs. 90%) and DNA‐protective (95% vs. 90%) properties (Lin et al. 2022). Notably, even with similar antioxidant indices, geographical origin, and extraction methodology seem to be the determining factors of variability in reported efficacy rather than the intrinsic differences in species, thus highlighting the need for standardized comparative studies.

## 7 Future Research Priorities

Instead of being limited to therapeutic development, future research on medicinal plants needs to be more rigorous and systematic in integrating different approaches. Future research in medicinal plants should be primarily concerned with isolating and characterizing compounds using bioassay‐guided systematic fractionation, thus identifying newer potential bioactive compounds. At the same time, other Mechanistic Studies using bio‐omic approaches will provide more detail on the molecular mechanisms of specific target(s) and/or downstream pathways of interest. Pharmacokinetic Studies: Absorption, Distribution, Metabolism, and Excretion (ADME) of specific compounds need to be done to determine the effective dosage to be administered. In the absence of this preclinical work, Clinical Validation may be limited to randomized controlled studies. Controlled studies will be of limited value to us if Formulation Development does not focus on the creation of more sophisticated delivery systems to improve efficacy. In the context of Comparative Phytochemistry, less metabolomic profiling will be of value if more sophisticated systems to monitor and report the metabolomic state are not developed. Finally, Defining Sustainable Cultivation will be more of a priority in the future to determine how best to obtain as much high‐grade plant material as possible for research and for future therapeutics.

## 8 Conclusion

This review comprehensively consolidates the phytochemical diversity and pharmacological potential of *F. benghalensis* , highlighting its validated antioxidant, antimicrobial, antidiabetic, anti‐inflammatory, and anticancer activities across multiple experimental models. The plant's rich repertoire of bioactive constituents, including flavonoids, terpenoids, sterols, phenolic acids, and unique glycosides, underpins its multi‐targeted mechanisms such as free radical scavenging, inhibition of α‐amylase, α‐glucosidase, PTP1B, COX‐1/COX‐2, 5‐LOX, and topoisomerase I, modulation of EGFR/PI3K/Akt signaling, induction of apoptosis, and G_2_/M cell cycle arrest. Quantitative in vitro and in vivo findings consistently demonstrate significant bioactivity, while emerging nanoformulation approaches suggest promising strategies to enhance bioavailability and therapeutic selectivity. Despite strong preclinical evidence, the clinical translation of *F. benghalensis* remains limited by the lack of standardized extracts, insufficient pharmacokinetic and toxicological profiling, and the absence of controlled human studies. Addressing these gaps through bioassay‐guided isolation, rigorous ADME and safety evaluations, standardized formulation development, and well‐designed clinical trials will be critical to advancing *F. benghalensis* from traditional medicine to evidence‐based phytopharmaceutical applications for inflammatory, metabolic, and oncological disorders.

## Author Contributions

**Zainab Ali:** writing – original draft, writing – review and editing, conceptualization, visualization, validation. **Iffat Ullah:** formal analysis, conceptualization, writing – review and editing, investigation, writing – original draft. **Farhang Hameed Awlqadr:** writing – review and editing, formal analysis, investigation, conceptualization, writing – original draft. **Uzma Faridi:** writing – review and editing, writing – original draft, formal analysis, project administration, supervision. **Muhammad Tayyab Arshad:** data curation, writing – review and editing, writing – original draft, investigation, visualization. **Sayeed Mukhtar:** writing – review and editing, writing – original draft, investigation, validation, visualization. **Md. Sakhawot Hossain:** validation, writing – review and editing, writing – original draft, formal analysis, conceptualization. **Uswa Ali:** writing – review and editing, writing – original draft, formal analysis, supervision, resources. **Abdul Rauf:** supervision, methodology, writing – review and editing, writing – original draft, resources. **Humaira Parveen:** writing – review and editing, writing – original draft, conceptualization, visualization, supervision.

## Funding

This authors have nothing to report.

## Ethics Statement

The authors have nothing to report.

## Consent

This study did not involve human or animal subjects.

## Conflicts of Interest

The authors declare no conflicts of interest.

## Data Availability

The data that support the findings of this study are available from the corresponding author upon reasonable request.
